# Investigation of fluoride evaporation from CaF_2_–CaO–Al_2_O_3_–MgO–TiO_2_–(Li_2_O) slag for electroslag remelting

**DOI:** 10.1038/s41598-020-69283-6

**Published:** 2020-07-23

**Authors:** Jiantao Ju, Guangheng Ji, Chenmei Tang, Kangshuai Yang, Zhihong Zhu

**Affiliations:** 1 School of Metallurgical Engineering, Xi’an University of Architecture and Technology, Xi’an, 710055 People’s Republic of China; 2 Research Center of Metallurgical Engineering Technology of Shaanxi Province, Xi’an, 710055 People’s Republic of China

**Keywords:** Chemical physics, Theory and computation

## Abstract

The isothermal kinetics of fluoride evaporation from CaF_2_–CaO–Al_2_O_3_–MgO–TiO_2_–(Li_2_O) slag with varying Li_2_O content were investigated in the temperature range 1743–1803 K by thermogravimetric analysis. Thermodynamic calculations and viscosity measurements were applied for studying the evaporation mechanism of fluoride. The results showed that the evaporation ratio increases with increasing Li_2_O content and temperature. The volatile constituents from the molten slags, mainly LiF and CaF_2_, were detected and their concentrations calculated. The fluoride evaporation is primarily affected by the vapour pressure of LiF and CaF_2_, viscosity of the slags, and melt-component activities under given experimental conditions. On the other hand, mass transfer of the gas is not the rate-controlling step that affects fluoride evaporation from the slags. The activation energy for fluoride evaporation gradually decreased from 193 ± 11 to 113 ± 3 kJ mol^−1^ as the Li_2_O content in the slags increases from 0 to 5.48 wt%. These results hold great theoretical significance for developing low-fluoride slags for electroslag remelting.

## Introduction

The slags in electroslag remelting (ESR) process serve as a heat source, a barrier to the atmosphere, and a medium for liquid metals refining^1–5^. To perform the intended functions, the slags must have some well-defined properties such as low electrical conductivity and suitable viscosity at remelting temperature^6,7^. The traditional ESR slags are CaF_2_–CaO–Al_2_O_3_-based slags with a high CaF_2_ content, and an additive such as MgO, TiO_2_ or SiO_2_ is commonly used to meet specific material requirements. However, fluoride evaporation from high CaF_2_-content slags is a potential health- and safety hazard. Furthermore, it would alter the chemical composition and cause changes in the metallurgical properties of the slags^8–11^. Therefore, it is important to develop low-fluoride slags and study their effect on fluoride evaporation during the ESR process.

The most common approach for developing low-fluoride slags is to control the CaO/Al_2_O_3_ mass ratio and substitute a small amount of oxides for CaF_2_ to keep the physicochemical properties stable. Several oxides including Na_2_O, B_2_O_3_, and Li_2_O are considered as potential substitutes for CaF_2_^12–14^. Among them, a small addition of Li_2_O could effectively regulate the viscosity and melting temperature of the slags; thus, the partial substitution of CaF_2_ with Li_2_O retains the performance of the slags. Several works reported by Shi et al*.*^15^ have researched the effect of Li_2_O on the viscosity of CaF_2_–CaO–Al_2_O_3_–MgO slags, which suggested that the viscosity of slags decreased with Li_2_O content increasing from 0 to 4.5 wt%. Kim et al*.*^16^ measured the viscosity of CaO–Al_2_O_3_-12 wt% Na_2_O-12 wt% CaF_2_-based slags and concluded that the addition of Li_2_O depolymerized the large aluminate structures and decreased the slag viscosity. Additionally, Liu et al.^17^ studied the effect of Li_2_O on the properties of CaF_2_–CaO–Al_2_O_3_–SiO_2_–Na_2_O–Li_2_O slags, and discovered that adding a small account of Li_2_O could decrease the slag-melting temperature. The characteristic temperature experiments conducted by Qi et al.^18^ indicated that the melting temperature of CaO–Al_2_O_3_–Li_2_O–Ce_2_O_3_ slags reduced gradually with the increase of Li_2_O content. In conclusion, Li_2_O provides an optimum condition for reducing the viscosity and melting temperature. Thus, it could be selected as an effective component to design the low-fluoride slags for ESR. However, several studies have reported that Li_2_O could react with CaF_2_ resulting in the appearance of gaseous LiF^19,20^. Such a loss of the fluoride will lead to serious environmental pollution and health problems^21,22^. However, the previous studies are very limited in demonstrating the mechanism of fluoride evaporation. Thus, it is necessary to study the effect of Li_2_O on the fluoride evaporation from low-fluoride slags.

In the present work, isothermal thermogravimetry was employed to investigate the kinetics of fluoride evaporation from CaF_2_–CaO–Al_2_O_3_–MgO–TiO_2_–(Li_2_O) slags containing 0–6 wt% Li_2_O in the temperature range 1743–1803 K. Meanwhile, the evaporation of fluoride was qualitatively analysed using thermodynamic calculations and viscosity measurements. The influence of Li_2_O on the mechanism of fluoride evaporation was clarified to gain the theoretical understanding of the development of low-fluoride slags for ESR.

## Materials and methods

### Preparation of slag samples

**Figure 1:**
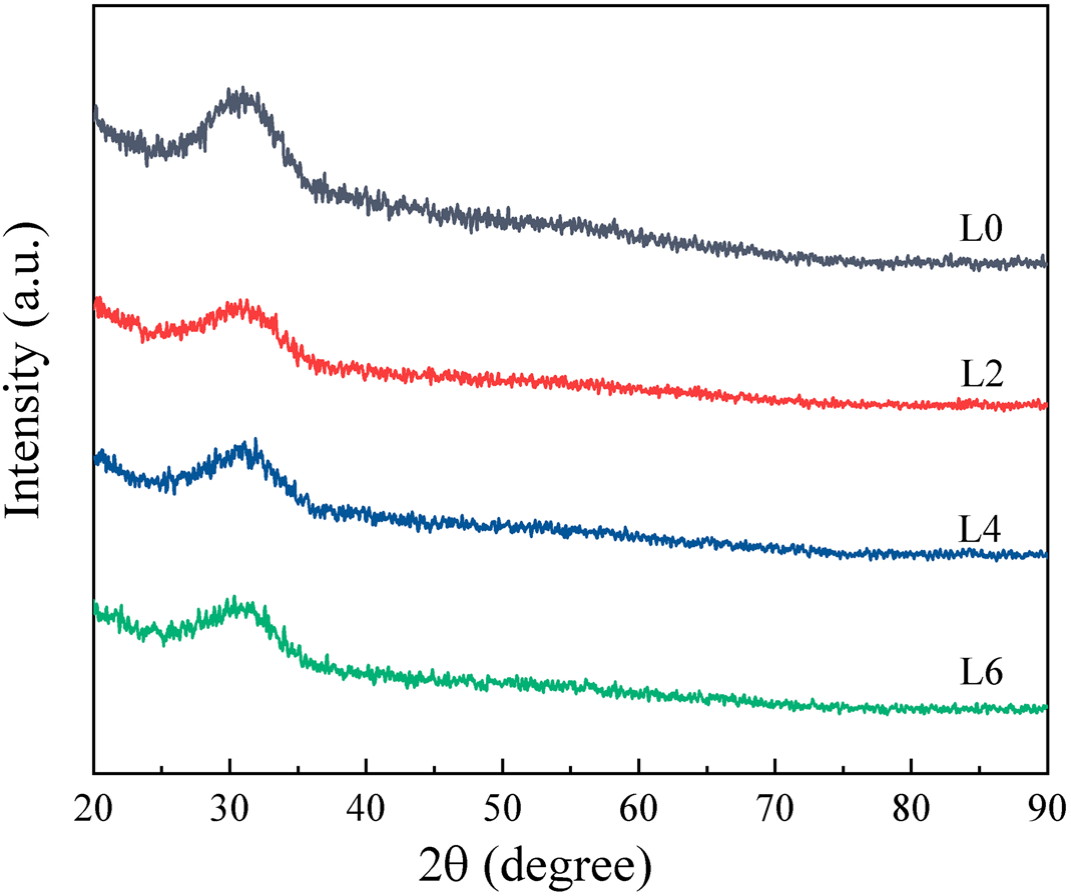
XRD patterns of the pre-melted samples.

All slag samples were prepared from analytical-grade reagents of CaF_2_ (≥ 98.5%), CaO (≥ 98.0%), Al_2_O_3_ (≥ 99.0%), MgO (≥ 98.0%), TiO_2_ (≥ 99.0%), and Li_2_O (≥ 99.9%). The initial compositions are listed in Table 1. A Pt crucible was filled with 50 g of the mixed powders and then placed in an electric resistance furnace at 1773 K for 10 min under high-purity Ar gas (> 99.999%) atmosphere to promote powder-composition homogeneity. The pre-melted samples were used for chemical analysis, thermogravimetric experiments, and viscosity measurements after crushing, grinding, and screening. The compositions of the pre-melted samples were confirmed by X-Ray fluoroscopy (XRF, Rigaku ZSX Primus II, Japan). The Li-content of the slags was analysed using inductively coupled plasma atomic emission spectroscopy (ICP-AES, Optima 7300 DV, Perkin Elmer, Waltham, MA, USA) with a charge-coupled device (CCD) detector, which was tuned before analysis as per the manufacturer’s recommended protocol. The uncertainty associated with the ICP-AES equipment was within ± 0.2%. The final compositions of the samples are also shown in Table 1. The pre-melted samples were verified to be amorphous by X-ray diffraction (XRD, D8 Advance, Bruker, Billerica, MA, USA, radiation source: Cu-Kα, tube voltage: 40 kV, and tube current: 40 mA) analysis, which is presented in Fig. 1.

**Table 1:** The compositions of the slag samples (wt%).

Slag samples	Initial composition						Final composition					
	CaF_2_	CaO	Al_2_O_3_	MgO	TiO_2_	Li_2_O	CaF_2_	CaO	Al_2_O_3_	MgO	TiO_2_	Li_2_O
L0	23.0	31.8	35.2	2.0	8.0	0	20.94	37.7	31.36	1.85	8.15	0
L2	23.0	30.8	34.2	2.0	8.0	2.0	20.48	37.3	30.55	1.83	8.19	1.65
L4	23.0	29.8	33.2	2.0	8.0	4.0	19.75	37.15	29.43	1.89	8.16	3.62
L6	23.0	29.0	32.0	2.0	8.0	6.0	19.6	36.55	28.18	1.86	8.33	5.48

### Experimental procedure

**Figure 2:**
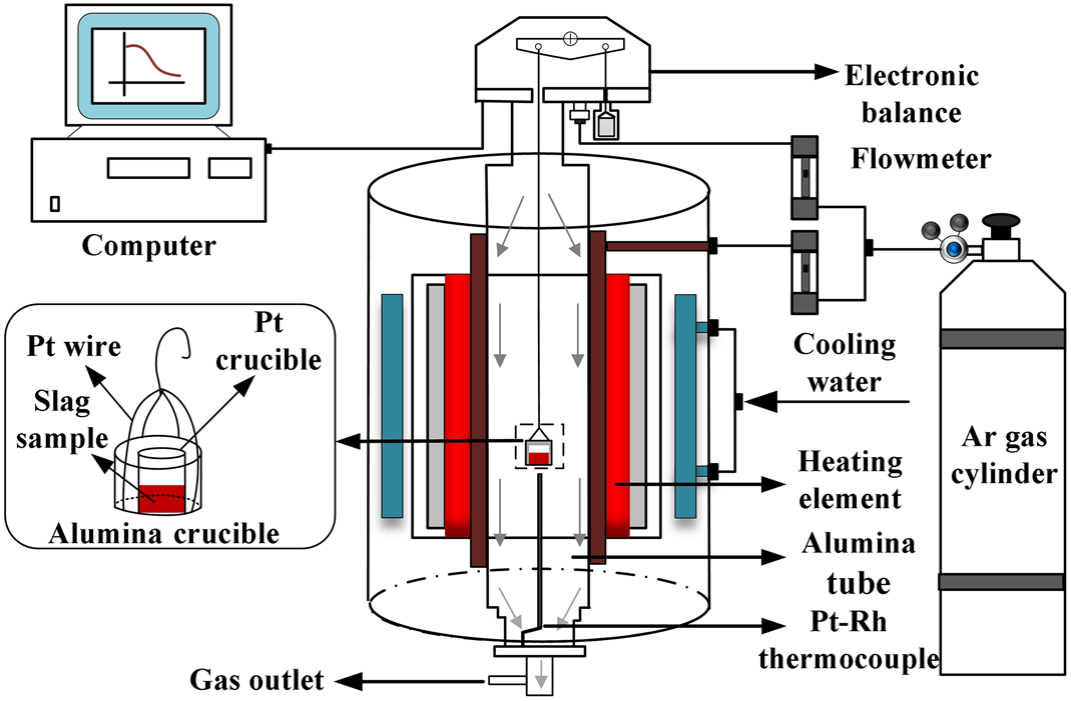
A schematic diagram of the experimental apparatus for thermogravimetric experiments.

Isothermal thermogravimetric experiments were conducted using a thermal analyser (Setsys EVO, Setaram instrument, France) having an accuracy of 0.2 μg at three temperatures: 1743, 1773, and 1803 K. Figure 2 shows a schematic of the experimental apparatus employed.

**Figure 3:**
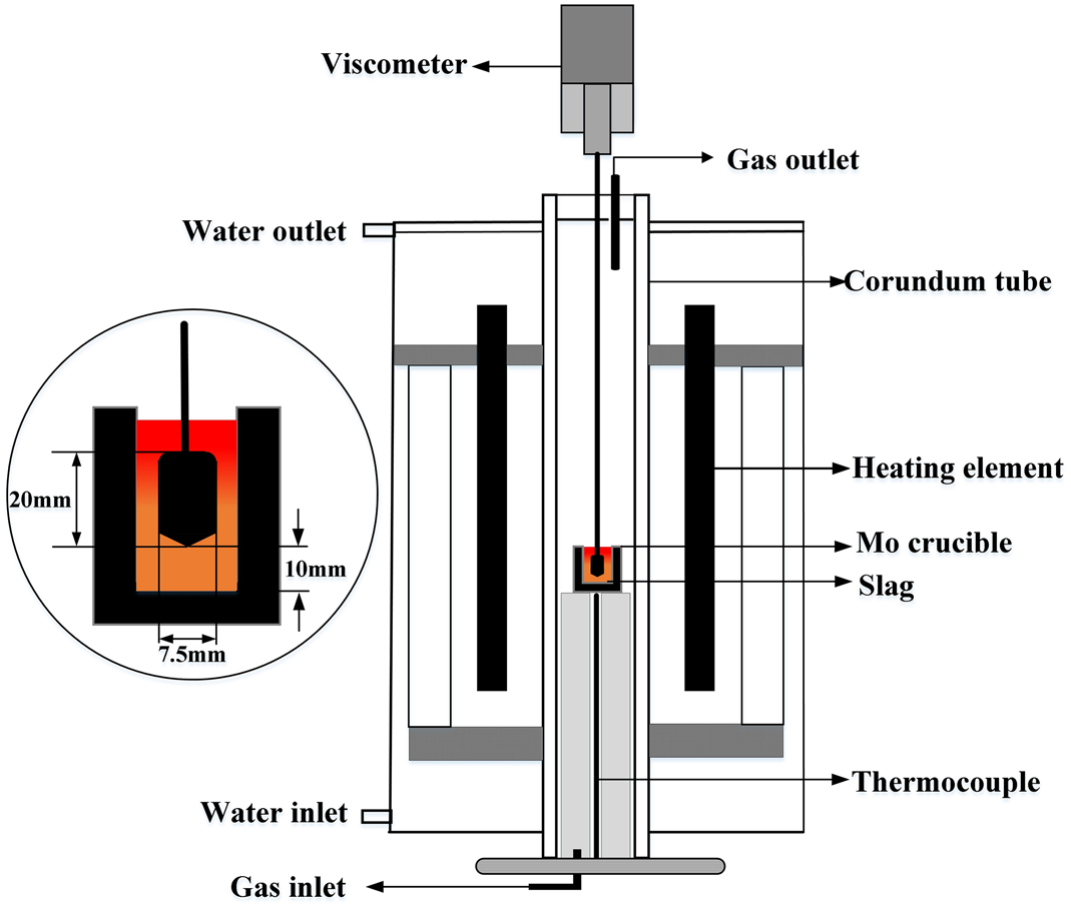
A schematic diagram of the experimental apparatus for viscosity measurements.

The experiments were conducted using the pre-melted samples weighting 15 ± 0.5 mg and having a particle size less than 74 μm. The slag samples were tested in a Pt crucible having inside diameter of 5.0 mm and height of 6.0 mm and under high-purity Ar gas with a flow rate of 70 mL min^−1^. To reduce the evaporation during the heating process, the samples were heated to the desired temperature at 100 K min^−1^ rate. The samples were held at the target temperature for 60 min. The Pt crucible was placed in an alumina crucible, which was suspended in the sample chamber and connected to the balance of the thermal analyser using a Pt wire. The length of the Pt wire was adjusted to ensure that the slags were positioned in the uniform-temperature zone. To exclude the influence of system error in the thermal analyser and buoyancy force of the gas mixtures, blank tests were conducted with empty crucibles. The slag viscosity measurements were conducted using a rotating spindle viscometer (HRV-1600P, Sinosteel Luoyang Institute of Refractories Co., Ltd, China), as shown in Fig. 3. The Mo crucible filled with 140 g pre-melted slag was placed in the uniform temperature zone of an electric resistance furnace. The experimental temperatures were controlled by a B-type (Pt-30% Rh-Pt-6% Rh) thermocouple inserted into the furnace with temperature fluctuation less than ± 2 K. The viscosity was measured at every 10 K during the cooling from 1823 to 1573 K and under high-purity Ar gas atmosphere.

Moreover, the thermodynamic calculations were performed with the principle of minimising Gibbs free energy using FactSage software (GTT Technologies, Aachen, Germany and Thermfact/CRCT, Montreal, QC, Canada)^23^. In the present work, the equilibrium module was used to predict the evaporating species. For the calculations, the equilibrium module was employed together with the databases FToxid and FactPS. Additionally, to understand the vapour pressures of volatile fluoride from the thermodynamics perspective, the equilibrium constant (*K*) and the component activity in the molten slags (*a*) were calculated using the FactSage software’s reaction module and equilibrium module, respectively.

## Results and discussion

### Analysis of weight loss process

The ratio of weight loss to the initial mass (α) is calculated from the thermogravimetric data by Eq. (1)

1 $$ \alpha = \frac{{m_{0} - m_{t} }}{{m_{0} }} $$

**Figure 4:**
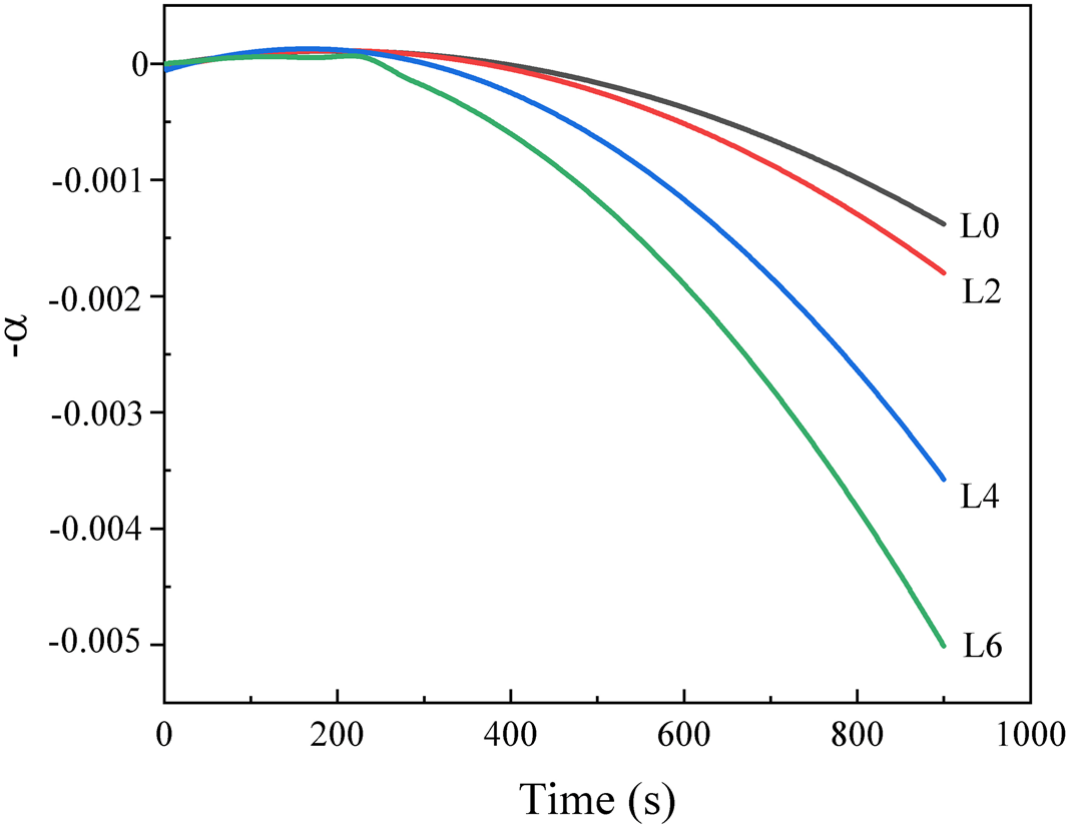
Weight loss of the slags during the heating process.

Here, *m*_*0*_ represents the initial slag mass. *m*_*t*_ is the slag mass at time t. Figure 4 shows the weight loss of the slags during the heating process, which is very small (< 0.5%) and hence can be ignored.

**Figure 5:**
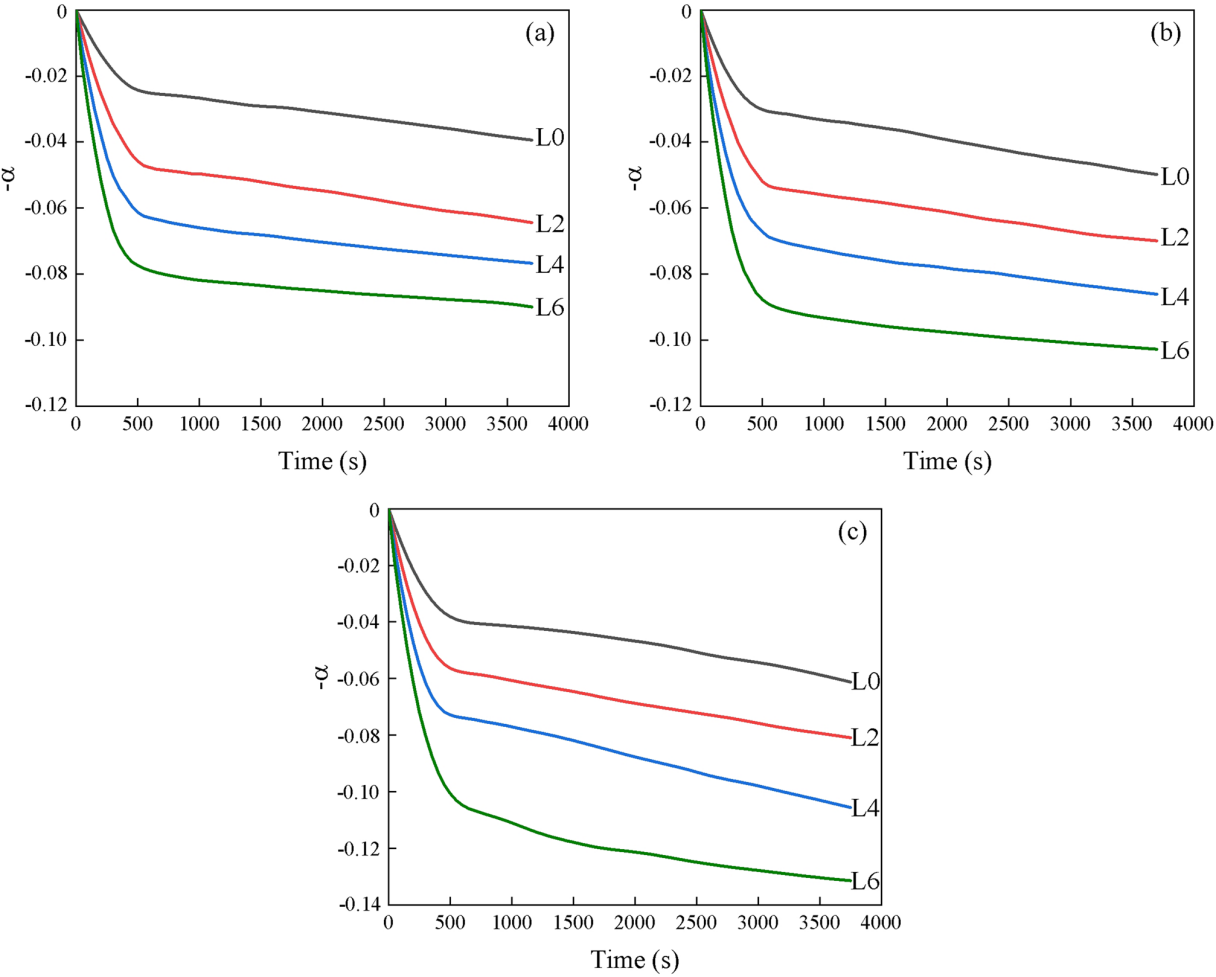
Comparison of the weight loss of the slags at different temperatures: (**a**) 1743, (**b**) 1773, and (**c**) 1803 K.

**Figure 6:**
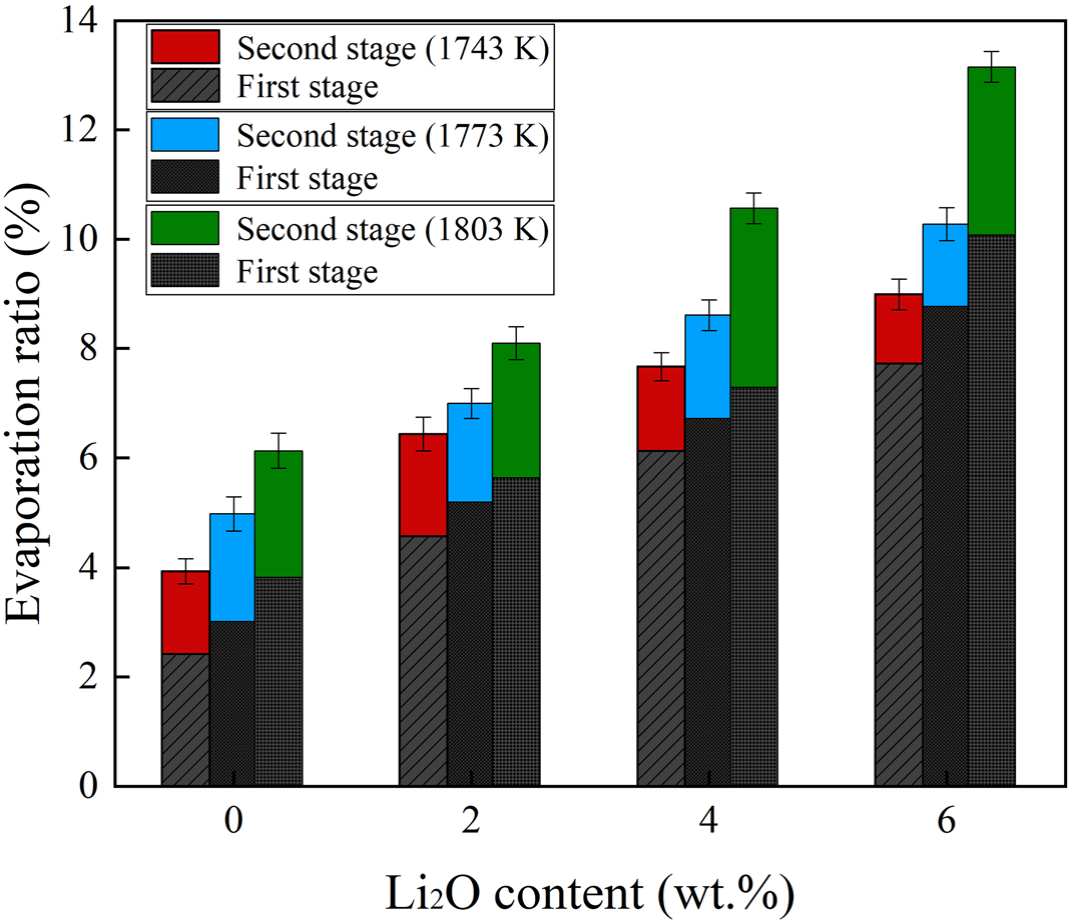
The evaporation ratio of the slags at different temperatures.

The weight loss curves of the slags at different temperatures are shown in Fig. 5a–c. The weight loss is mainly attributed to the fluoride evaporation at high temperatures. The chemical composition and temperature have significant influence on the weight loss of the slags. The maximum values of α of L0, L2, L4, and L6 at 1743 K are 3.94, 6.44, 7.67, and 9.00%, respectively. The α value obviously increases with increasing Li_2_O content. The detailed discussion on the effect of Li_2_O on evaporation will be discussed in the next section. The maximum values of α at 1773 and 1803 K increase from 4.98 to 10.28% and 6.13 to 13.15%, respectively, when Li_2_O content increases from 0 to 5.48 wt%. The results suggest that α gradually increases with increasing temperature. From kinetic point of view, an increase in temperature decreases the viscosity, which accelerates the liquid phase mass transport. The weight loss process of the slags can be separated into two stages. The evaporation ratio rapidly increases in the first 500 s, and shows a slower increase at longer times. Figure 6 shows the evaporation ratio in two stages. It should be noted that the evaporation from the molten slags mainly occurs in the first stage.

## Kinetics of evaporation

### Evaporated gaseous species

The possible reactions occurring during the isothermal experiments are as follows:

2 $$ \left( {{\text{CaF}}_{{2}} } \right)_{{{\text{slag}}}} = {\text{ CaF}}_{{2}} \left( {\text{g}} \right) $$

3 $$ \left( {{\text{CaF}}_{{2}} } \right)_{{{\text{slag}}}} + \, \left( {{\text{Li}}_{{2}} {\text{O}}} \right)_{{{\text{slag}}}} = \, \left( {{\text{CaO}}} \right)_{{{\text{slag}}}} + {\text{ 2LiF}}\left( {\text{g}} \right) $$

4 $$ \left( {{\text{CaF}}_{{2}} } \right)_{{{\text{slag}}}} + \, \left( {{\text{MgO}}} \right)_{{{\text{slag}}}} = \, \left( {{\text{CaO}}} \right)_{{{\text{slag}}}} + {\text{ MgF}}_{{2}} \left( {\text{g}} \right) $$

5 $$ {3}\left( {{\text{CaF}}_{{2}} } \right)_{{{\text{slag}}}} + \, \left( {{\text{Al}}_{{2}} {\text{O}}_{{3}} } \right)_{{{\text{slag}}}} = { 3}\left( {{\text{CaO}}} \right)_{{{\text{slag}}}} + {\text{ 2AlF}}_{{3}} \left( {\text{g}} \right) $$

6 $$ \left( {{\text{CaF}}_{{2}} } \right)_{{{\text{slag}}}} + {\text{ AlF}}_{{3}} \left( {\text{g}} \right) \, + \, \left( {{\text{CaO}}} \right)_{{{\text{slag}}}} = {\text{ 2CaF}}_{{2}} \left( {\text{g}} \right) \, + {\text{ AlOF}}\left( {\text{g}} \right) $$

**Figure 7:**
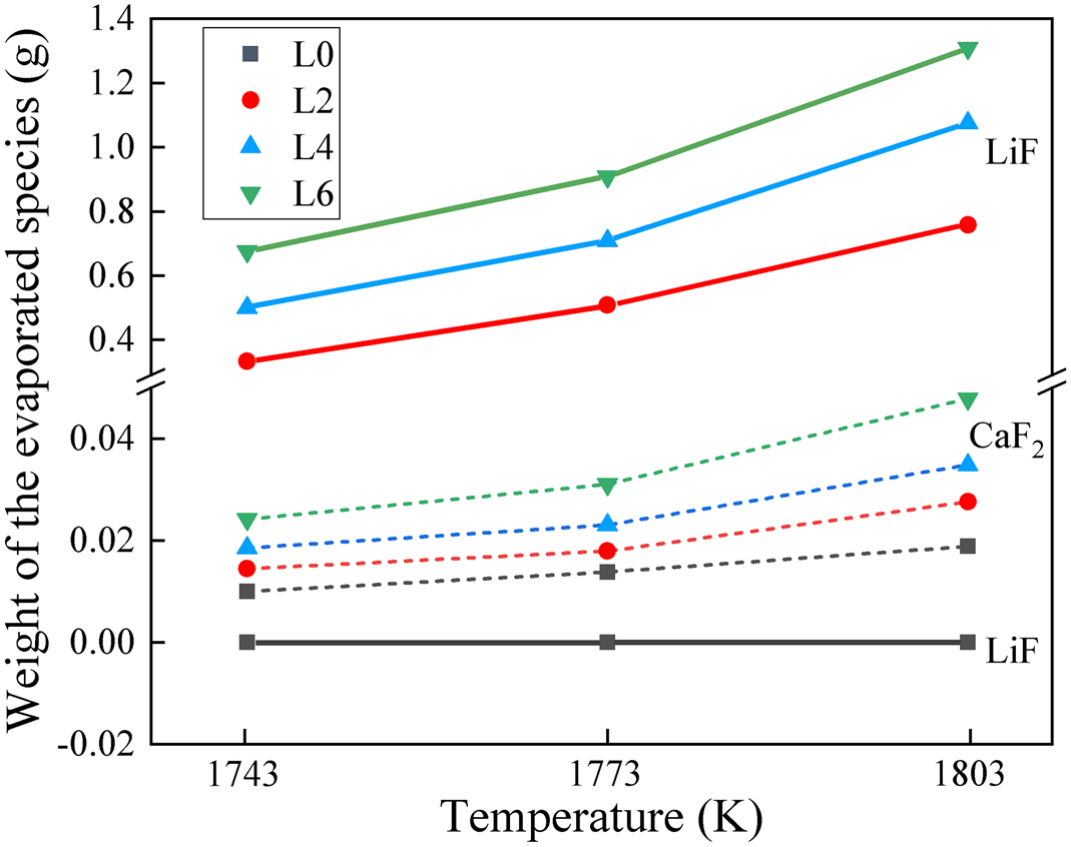
Weight of the gaseous species evaporating from the slags at different temperatures.

The gaseous species evaporating from the molten slags at different temperatures were calculated using FactSage software. The results are shown in Fig. 7. Among them, the volatile constituents were identified (LiF and CaF_2_) and their quantities were calculated. The estimated weight of LiF was obviously greater than that of CaF_2_. The quantities of gaseous MgF_2_, AlF_3_, and AlOF generated were negligible. Therefore, Eqs. (2) and (3) would be expected to play a major role in the evaporation process. Similar results were obtained by Zheng et al*.*^19^ showing that the gaseous species evaporating from the CaF_2_–CaO–Al_2_O_3_–MgO–Li_2_O slag melts were mainly LiF and contained a small amount of CaF_2_ at various temperatures. Meanwhile, the results revealed that both the temperature and chemical composition of the slag affect the fluoride evaporation. The weight of LiF and CaF_2_ evaporating from the molten slags increased with increasing temperature as well as Li_2_O content.

### Rate-controlling step

**Figure 8:**
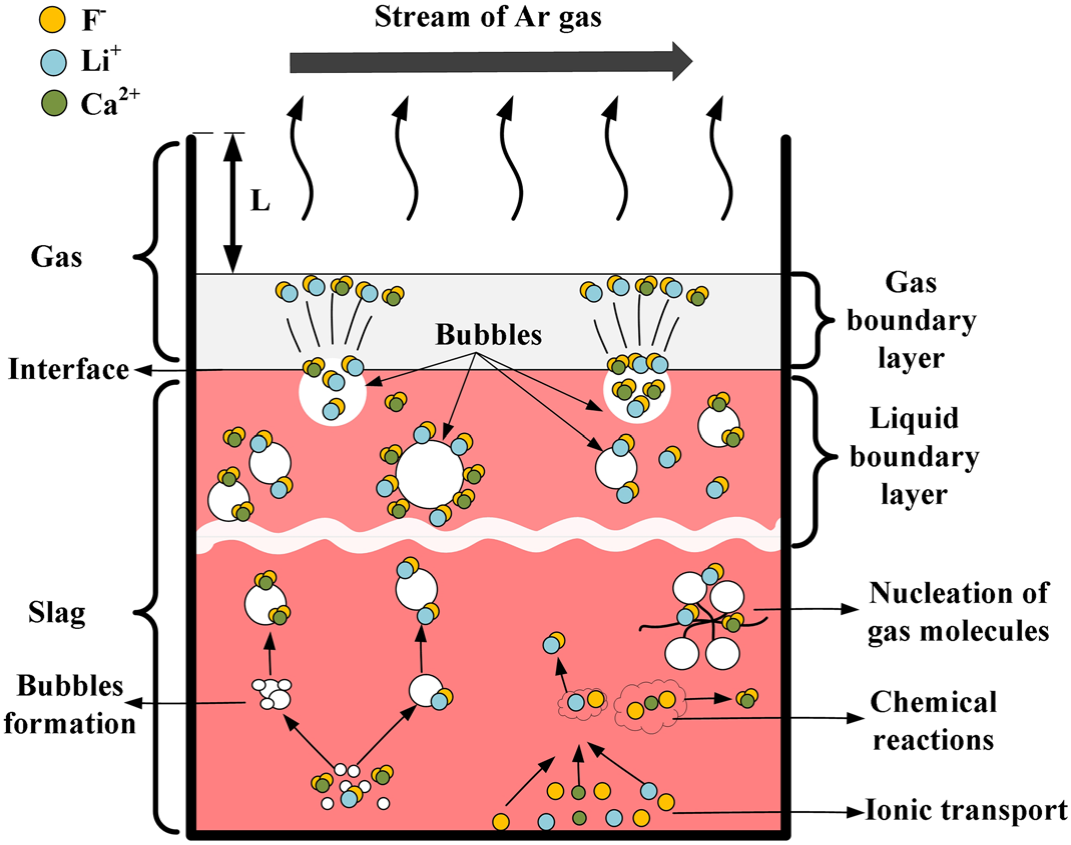
The schematic model of the fluoride evaporation mechanism.

The rate-controlling step of the fluoride evaporation process can be one or a combination of the following steps (Fig. 8)^24–26^:

1. Mass transfer in the liquid slag. The transport of the anion (F^-^) and cations (Li^+^ and Ca^2+^) involved in Eqs. (2) and (3) to the reaction site.
2. Chemical reactions Eqs. (2) and (3).
3. Nucleation of LiF and CaF_2_ gas molecules.
4. The transport of bubbles from the bulk liquid slag to the slag/gas interface through the liquid boundary layer.
5. The transport of the gas from the slag/gas interface to the bulk gas flow through the stagnant gas film.
6. Flow of the bulk gas stream from the crucible.

The vapour pressures of the fluoride gases reflect the driving force for the corresponding evaporation reactions, which determine the yields and rates of the corresponding chemical reactions^27^. The equilibrium constants for Eqs. (2) and (3) can be expressed as follows:

7 $$ K_{Eq.(2)} = \frac{{a_{(CaO)} \cdot (P_{LiF} )^{2} }}{{a_{{(CaF_{2} )}} \cdot a_{{(Li_{2} {\text{O}})}} }} $$

8 $$ K_{{Eq.(3)}}  = \frac{P_{CaF_{2}}}{a_{(CaF_{2})}} $$

Here, *P*_*LiF*_ and *P*_*CaF2*_ represent the vapour pressures (atm.) of LiF and CaF_2_, respectively, and *a*_*i*_ is the activity of the melt component *i* in the molten slags. *K*_*Eq*_ and *a*_*i*_ were calculated by thermodynamic calculations of FactSage software. The vapour pressures of LiF and CaF_2_ were calculated by entering the equilibrium constant values and activity data into Eqs. (7) and (8); the results are plotted in Table 2. The vapour pressures were similar for identical gaseous species at a given temperature, while the vapour pressures of the evaporating species increased with increasing temperature. The vapour pressure of LiF was much higher than that of CaF_2_ in lithium-containing slags. When gaseous species with different vapour pressures exist, the species with higher vapour pressure vaporise preferentially^14,28^. Consequently, LiF is the major component that leads to the fluoride loss in Li_2_O-containing slag, and CaF_2_ is the main volatile product in the L0 slag. These results are consistent with those from the thermodynamic calculations. It can be obtained by calculating vapour pressures that chemical reactions Eqs. (2) and (3) [step (2) of the proposed mechanism] are important factors for affecting evaporation of the slags.

**Table 2:** The calculated vapour pressures of LiF and CaF_2_ (10^–6^ atm).

Vapour pressure	T(K)	L0	L2	L4	L6
*P*_LiF_	1743	0	22,375.6	22,375.4	22,375.5
	1773	0	29,317.4	29,317.4	29,317.4
	1803	0	38,024.8	38,025.0	38,025.1
*P*_CaF2_	1743	14.6	14.3	14.1	14.0
	1773	22.0	21.6	21.3	21.0
	1803	30.0	32.2	31.6	31.1

**Figure 9:**
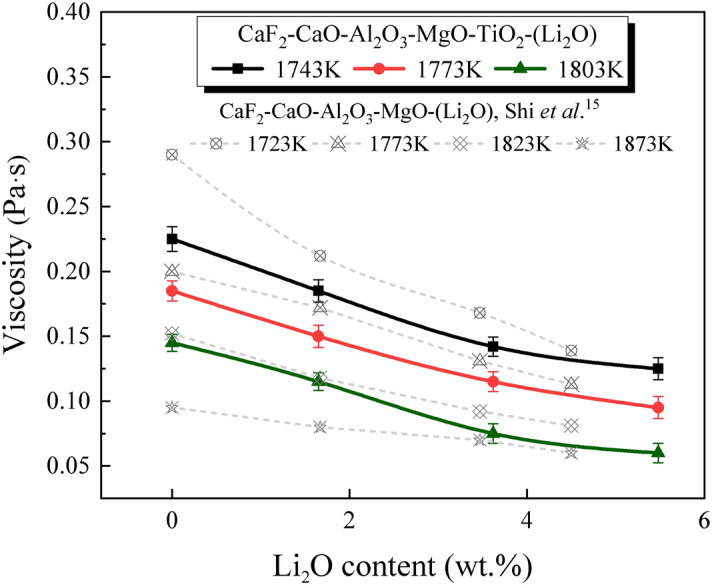
The viscosity of the slags at different temperatures.

The fluoride evaporation process is also affected by the viscosity and component activities of the slag^29,30^. The viscosity affects mass transfer and is closely related to the variation of slag structure^31,32^. The influence of Li_2_O content on the viscosity of the slags in the temperature range 1743–1803 K can be seen in Fig. 9. The viscosity is in 0.225–0.06 Pa·s range at temperatures 1743–1803 K, and it gradually decreases with increasing Li_2_O content and temperature This is similar to the work reported by Shi et al.^15^, which measured the viscosity of CaF_2_–CaO–Al_2_O_3_–MgO–Li_2_O slag. According to Park et al.^33^ and Neuville et al.^34^, under the silicate-free conditions, Al_2_O_3_ tends to form [AlO_4_]-tetrahedral units that consist of four oxygen atoms. These units combine to form complex aluminate structures, which increase the resistance to liquid mass transfer within the molten slag. Shi et al.^15^ and Kim et al.^16^, studied the effect of Li_2_O on the aluminate structure and the viscosity of the slags in the absence of silicates. They noted that Li_2_O acted as a network modifier i.e., it provided free oxygen (O^2−^) in the molten slags. The O^2−^ can interact with the bridged oxygen (O^0^) of the aluminates, leading to the depolymerisation of the large aluminate network into simpler structure. Thus, the addition of Li_2_O lowers the slag viscosity, leading to improved kinetic conditions for the fluoride evaporation. The lower viscosity also promotes liquid mass transfer and the transport of bubbles from bulk liquid slag to the slag/gas interface through the liquid boundary layer [steps (1) and (4) of the proposed mechanism].

The thermodynamic calculations of the melt component activities were performed using FactSage software according to the work reported by Zheng et al.^19^ The Li_2_O activity values calculated for various temperatures are listed in Table 3. Within the experimental temperature range, the Li_2_O activity of gradually increases with increasing Li_2_O content, which favours the formation of LiF. Moreover, increasing the temperature can promote liquid mass transfer and the transport of bubbles from bulk liquid slag to the slag/gas interface through the liquid boundary layer. Hence, σ increases with increasing temperature and Li_2_O content.

**Table 3:** The Li_2_O activity calculated for different temperatures by FactSage software.

Slag samples	Li_2_O activity		
	1743 K	1773 K	1803 K
L0	0	0	0
L2	0.0482	0.0474	0.0466
L4	0.0665	0.0635	0.0614
L6	0.0748	0.0753	0.0784

By comprehensively analysing the vapour pressures, viscosity of the slags, and melts component activities, it can be concluded that step (1), (2), and (4) play a significant part in controlling the evaporation process. These results are in agreement with those reported by Liu et al.^27^.

**Figure 10:**
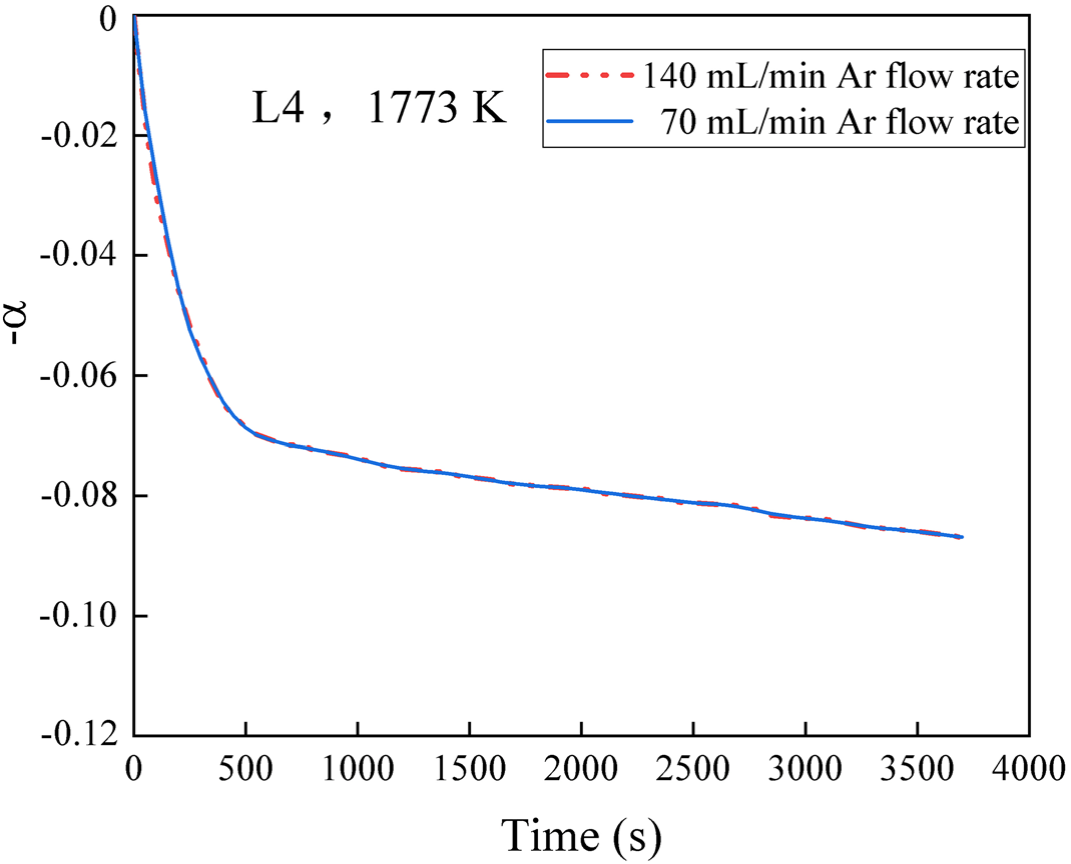
Weight loss of L4 slag at 1773 K at various Ar flow rates.

In the present study, step (5) and (6) are gas-mass transfer processes. In order to understand whether the Ar flow rate used (70 mL min^−1^) is above the starvation rate, the isothermal experiments were conducted under different Ar flow rates conducted. Figure 10 shows the weight loss of L4 slag at 1773 K with varying Ar flow rates. Changing the Ar flow rate from 70 to 140 mL min^−1^ did not appear to affect the fluoride evaporation rate in L4. This is because the bulk-gas flow rate of 70 mL min^−1^ is sufficient to carry the volatile constituents at a rate larger than that of the evaporation reaction itself. Hence, step (6) is unlikely to act the rate-controlling step.

In order to determine whether step (5) is rate-controlling step, the following analysis was performed based on the schematic model shown in Fig. 8. The Pt crucible was similar as the diffusion cell setup^26^. When fluoride evaporation starts, the gaseous species will evaporate from the interface and get transported out of the test apparatus by Ar gas. The mass concentrations of the gaseous species in the top part of the crucible are related to the Ar flow rate in the present experimental conditions. Considering that the bulk flow rate of Ar gas is much larger than the starvation rate, it can be assumed that the fluoride concentration at the top part of the crucible is extremity small or virtually zero. The fluxes of LiF and CaF_2_ vapours from the liquid surface are presented as follows:

9 $$ J_{LiF} = \frac{{D_{LiF} }}{L \cdot RT}(P_{LiF}^{i} - P_{LiF}^{b} ) $$

10 $$ J_{{{\text{Ca}}F_{2} }} = \frac{{D_{{{\text{Ca}}F_{2} }} }}{L \cdot RT}(P_{{{\text{Ca}}F_{2} }}^{i} - P_{{{\text{Ca}}F_{2} }}^{b} ) $$

Here, *D*_*LiF*_ and *D*_*CaF2*_ represent the diffusion coefficients of LiF and CaF_2_, respectively. through Ar gas (cm^2^ s^−1^). *L* is the distance between the surface of molten slag and the top edge of the crucible (cm). *R* is the gas constant [8.314 J/(mol K)]. *T* is the absolute temperature (K). *P*^*i*^ and *P*^*b*^ are vapour pressures at the slag/gas interface and in the bulk of the flow, respectively (atm). *P*^*b*^ is assumed zero under the present experiment. The diffusion coefficient is typically estimated using the Chapman–Enskog equation^35,36^

11 $$ D_{AB} = 0.0018583\sqrt {T^{3} \left( {\frac{1}{{M_{A} }} + \frac{1}{{M_{B} }}} \right)} \frac{1}{{P \cdot \sigma_{AB}^{2} \cdot \Omega_{AB} }} $$

Here, *D*_AB_ is the mass diffusivity of A through B (cm^2^ s); ^−1^σ_*AB*_, Ω_*AB*_ are the Lennard–Jones parameters, and the dimensionless quantity Ω_*AB*_ is the collision integral, which is function of dimensionless temperature (κ*T*/ε). *M*_*A*_ and *M*_*B*_ are molecular weights of component LiF or CaF_2_ and Ar gas, respectively. *P* is the absolute pressure (atm). The parameters for Ar gas are available from the literature. However, the experimental data is not available for specific gaseous species such as LiF and CaF_2_. Their parameters can be estimated by the following empirical relations^36^:

12 $$ \varepsilon /\kappa = 1.92T_{m} \;({\text{K}}) $$

13 $$ \sigma = 1.22V_{m}^{1/3} \;({\AA}) $$

Here, *T*_*m*_ is the melting temperature (K) and *V*_*m*_ is the molecular volume (cm^3^ mol^−1^). The values of ε/κ and σ for different gaseous species^26,37^ are shown in Table 4. The mixture parameters σ_*AB*_ and ε_AB_ were then estimated from Eqs. (14) and (15)^38^.

14 $$ \sigma_{AB} = \frac{1}{2}(\sigma_{A} + \sigma_{B} ) $$

15 $$ \varepsilon_{AB} = \sqrt {\varepsilon_{A} \varepsilon_{B} } $$

**Table 4:** The values of ε/κ and σ for different gaseous species.

Gas species	ε/κ (K)	σ (Å)	Molecular weight (g mol^−1^)	Melting temperature (K)	Molecular volume (cm^3^ mol^−1^)
Ar	122.4	3.4	40.0	–	75.2
LiF	2,152.3	3.0	25.9	1,121	14.4
CaF_2_	3,246.7	3.8	78.1	1691	30.6

The estimated diffusion coefficients of LiF and CaF_2_ in Ar gas were also calculated using Eq. (11), and the values are listed in Table 5. The diffusion coefficient of LiF in Ar gas is higher than that of CaF_2_ at a given temperature within the experimental range 1743–1803 K, which further confirms that the evaporation rate of LiF is higher than that of CaF_2_.

**Table 5:** Estimated diffusion coefficients of the gaseous species in Ar gas (cm^2^ s^−1^).

Item	1743 K	1773 K	1803 K
D(LiF-Ar)	0.98	0.99	1.00
D(CaF_2_-Ar)	0.72	0.73	0.74

The theoretical evaporation rates from the molten slags can be calculated using Eq. (16). A comparison of estimated and measured evaporation rates is shown in Table 6.

16 $$ \frac{dw}{{dt}} = M_{LiF} \cdot J_{LiF} + M_{{CaF_{2} }} \cdot J_{{CaF_{2} }} $$

**Table 6:** Comparison of the estimated and measured evaporation rates.

Slag samples	T(K)	Estimated rate [10^–6^ mg/(cm^2^ s)]	Measured rate [10^–6^ mg/(cm^2^·s)]
L0	1743	0.18	6.31
	1773	0.27	6.73
	1803	0.388	6.836
L2	1743	128.08	8.00
	1773	165.89	8.15
	1803	212.76	8.37
L4	1743	128.07	8.48
	1773	165.88	9.04
	1803	212.76	9.21
L6	1743	128.07	9.10
	1773	165.88	9.32
	1803	212.75	9.68

The estimated rates show a large deviation compared with the experimentally measured rates, and their difference is about an order of magnitude. Thus, step (5) is unlikely to be the rate-controlling step for the differences are too large. The findings of this research are consistent with previous studies by Li et al.^39^ and Tong et al.^40^ concerning the evaporation of NaBO_2_ and B_2_O_3_.

Based on the discussion above, step (5) and step (6) are not rate-limiting steps. However, steps (1), (2), step (4) would be the rate-controlling steps for the evaporation of LiF and CaF_2_ in the present experiment. Further studies are needed to determine whether step (3) is the rate-controlling process.

### Activation energy of evaporation

The activation energy of evaporation is a critical parameter to evaluate the propensity of evaporation. The Activation energy for evaporation can be determined by fitting the Arrhenius equation^41^

17 $$ \ln k = \frac{{ - E_{a} }}{RT} + \ln A $$

**Figure 11:**
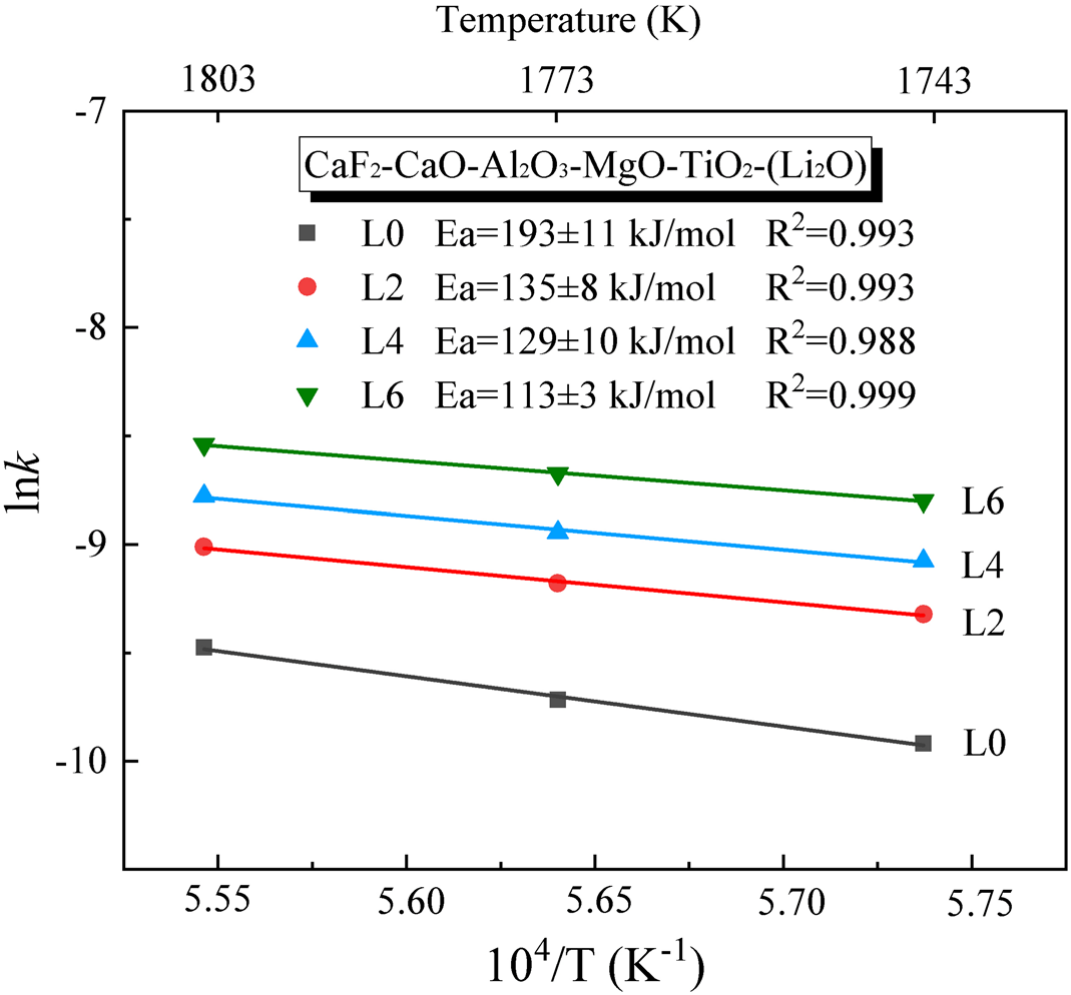
The activation energy for the evaporation of the slags with varying Li_2_O content.

Here, *k* represents the rate constant, *T* is the reaction temperature, *A* is the pre-exponential factor (s^−1^), and *E*_*a*_ is the activation energy (J mol^−1^). It is worth mentioning that the evaporation rates appear almost linear trend in four slag samples in the first stage. Since the chemical composition of the slags changes significantly due to fluoride evaporation, the present study took the initial 500 s to calculate the activation energy of evaporation. The activation energy for evaporation of the slags with varying Li_2_O contents is presented in Fig. 11. The correlation coefficient (R^2^) obtained from the data fitting procedure is close to 1. The calculated values of activation energy decreased with the addition of Li_2_O to the slags. The *E*_*a*_ value calculated for L0 was 193 ± 11 kJ mol^−1^, which gradually dropped to 113 ± 3 kJ mol^−1^ as the Li_2_O content increased. It further proves that Li_2_O, as a network modifier, depolymerizes the intricate network structure and thus decreases the activation energy of the evaporation process.

## Conclusions

The effect of Li_2_O on the fluoride evaporation of the CaF_2_–CaO–Al_2_O_3_–MgO–TiO_2_–(Li_2_O) slag was investigated in the temperature range 1743–1803 K using isothermal thermogravimetry. Our results are summarised as follows:

1. The fluoride evaporation from the molten slags occurred primarily during the initial 500 s; the process was promoted by increasing the Li_2_O content and temperature.
2. The species evaporating from the molten slags mainly consisted of LiF and CaF_2_. The activation energy for the fluoride evaporation decreased from 193 ± 11 to 113 ± 3 kJ mol^−1^ as Li_2_O content increased from 0 to 5.48 wt%.
3. The evaporation of fluoride was primarily affected by its vapour pressure, viscosity of the slags, and activity of Li_2_O in the slags. On the other hand, gas mass transfer was not the rate-controlling step under the present experimental conditions.
4. A small amount of Li_2_O can effectively regulate the viscosity of the slags and serve as an effective component in low-fluoride slags for ESR. However, harmful gaseous LiF evaporates from the molten slags when CaF_2_ is substituted with Li_2_O.

## Data Availability

The datasets generated during the current study are available from the corresponding author on reasonable request.
